# Perception and Evaluation of a Knowledge Transfer Concept in a Digital Health Application for Patients With Heart Failure: Mixed Methods Study

**DOI:** 10.2196/56798

**Published:** 2025-03-31

**Authors:** Madeleine Flaucher, Sabrina Berzins, Katharina M Jaeger, Michael Nissen, Jana Rolny, Patricia Trißler, Sebastian Eckl, Bjoern M Eskofier, Heike Leutheuser

**Affiliations:** 1 Machine Learning and Data Analytics Lab, Department Artificial Intelligence in Biomedical Engineering, Friedrich-Alexander-Universität Erlangen-Nürnberg, Carl-Thiersch-Str. 2b, Erlangen, 91052, Germany, 49 9131 8528990; 2 ProCarement GmbH, Forchheim, Germany; 3 Institute of AI for Health, Helmholtz Zentrum München - German Research Center for Environmental Health, Neuherberg, Germany

**Keywords:** health literacy, digital Literacy, user-centered design, digital health app, heart failure, mixed methods study, user centered deign, usability, patient engagement, mHealth app, development

## Abstract

**Background:**

Digital health education can enhance the quality of life of patients with heart failure by providing accessible and tailored information, which is essential for effective self-care and self-management.

**Objective:**

This work aims to develop a mobile health knowledge transfer concept for heart failure in a user-centered design process grounded in theoretical frameworks. This approach centers on enhancing the usability, patient engagement, and meaningfulness of mobile health education in the context of heart failure.

**Methods:**

A user-centered design process was employed. First, semistructured stakeholder interviews were conducted with patients (n=9) and medical experts (n=5). The results were used to develop a health knowledge transfer concept for a mobile health app for heart failure. This concept was implemented as a digital prototype based on an existing German mobile health app for patients with heart failure. We used this prototype to evaluate our concept with patients with heart failure in a study composed of user testing and semistructured patient interviews (n=7).

**Results:**

Stakeholder interviews identified five themes relevant to mobile health education: individualization, content relevance, media diversity, motivation strategies, and trust-building mechanisms. The evaluation of our prototype showed that patients value the adaptation of content to individual interests and prior knowledge. Digital rewards such as badges and push notifications can increase motivation and engagement but should be used with care to avoid overload, irrelevance, and repetition.

**Conclusions:**

Our findings emphasize the importance of tailoring mobile health education to the specific needs and preferences of patients with heart failure. At the same time, they also highlight the careful implementation of motivation strategies to promote user engagement effectively. These implications offer guidance for developing more impactful interventions to improve health outcomes for this population.

## Introduction

In 2019, heart failure affected more than 56 million people worldwide [1]. The diagnosis of heart failure entails severe health consequences for patients and significant financial resources for public health systems [2]. In light of an aging population, heart failure is becoming increasingly important in society and requires new approaches for prevention, management, and therapy to mitigate health impact [3,4].

Heart failure requires a high level of active patient involvement [5]. Self-monitoring of physiological parameters, medication intake, nutrition, and physical activity are crucial factors in preventing the hospitalizations of patients [6,7]. In this context, health literacy plays a major role since high levels of health literacy in patients with heart failure promote patient empowerment, self-care practices, and medication adherence [8-10]. Health literacy can be described as the capacity of a person to access and understand health-related information, place it in context, and use it responsibly to promote and maintain good health [11]. With a focus on heart failure and other cardiovascular diseases, The American Heart Association concludes that health literacy is crucial for effective treatments for cardiovascular diseases and acknowledges information technology as a potential path to improving health literacy [12]. In the long-term, low health literacy is associated with an increased risk for hospitalizations and death in patients with heart failure [13].

To effectively deliver information to patients and achieve learning effects, mobile health technology offers great potential. This applies to populations with low health literacy in particular [14-16]. As smartphone access has become widespread across various demographic groups, including those with lower socioeconomic status, they provide an accessible platform for delivering tailored health interventions to underserved populations [17,18]. Enhancing health literacy requires attention not only to disease-specific content but also to digital health literacy, empowering users to navigate health-related inquiries online effectively [14,19,20]. However, several barriers have been identified, including the access to apps, the readability of content as well as the usability of digital health services [14,21]. In the context of heart failure, evidence about the effects of mobile health for promoting health literacy is still limited. Allida et al identified no increase in heart failure knowledge through digital educational content and unclear evidence on self-efficacy, self-care, and health-related quality of life [22]. In contrast, several recent studies demonstrated positive effects on quality of life, hospitalization rates, and self-management abilities [23-25]. However, despite the potential benefits, these approaches lack the incorporation of theoretical frameworks for engaging, appealing, and effective knowledge transfer. Furthermore, patients with heart failure are hardly involved in the development process of mobile health interventions.

Thus, the aim of this work was to develop a concept for a sustainable and engaging knowledge transfer with a specific focus on the needs and preferences of patients with heart failure. Theoretical frameworks for mobile health knowledge transfer and the involvement of end-users throughout the design process build a strong foundation for an engaging, user-friendly, and effective mobile health intervention. A digital prototype served as a tangible representation of our concept, which we evaluated with patients with heart failure.

## Methods

### Study Design Overview

**Figure 1.**
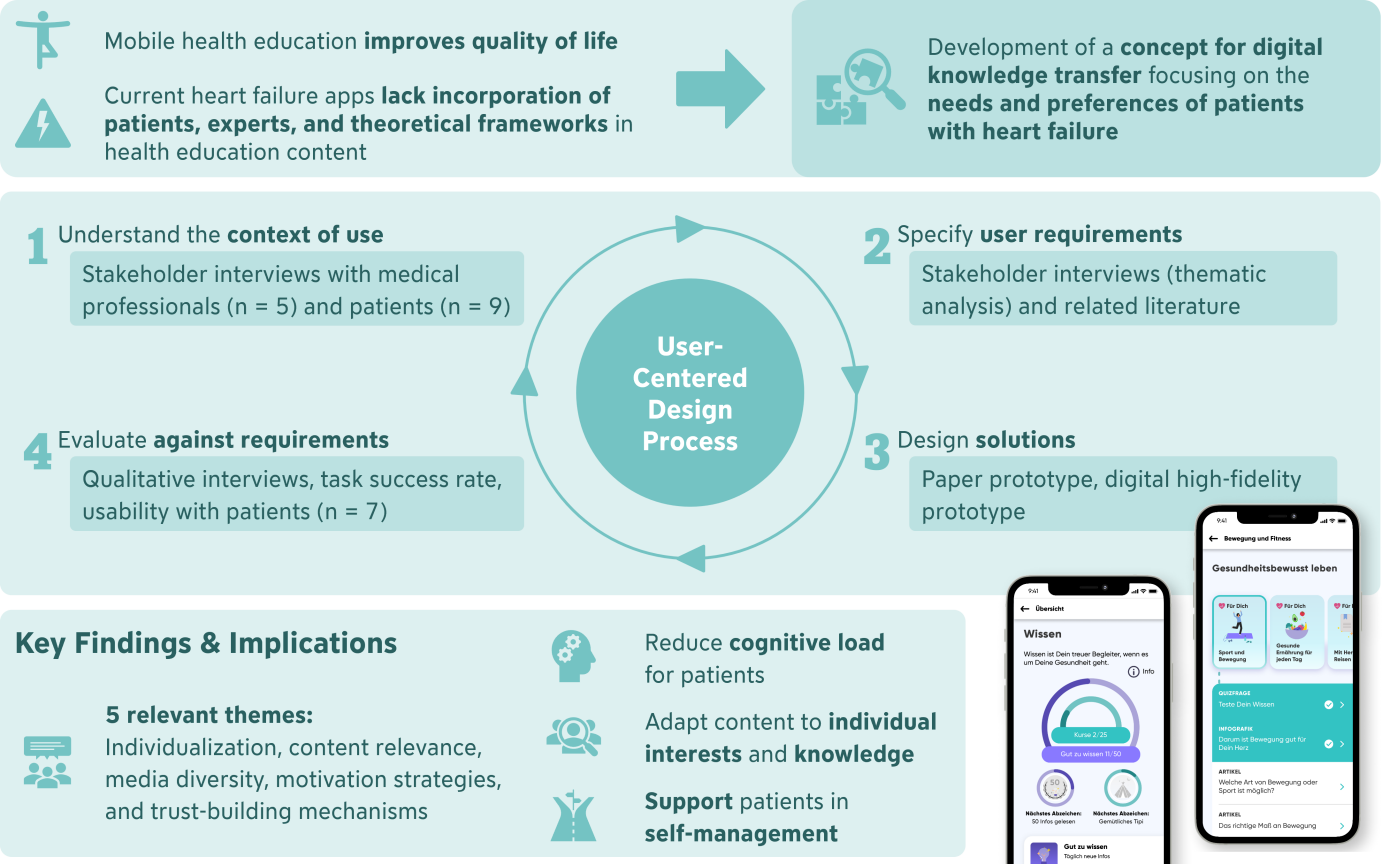
Graphical abstract illustrating the integration of the user-centered design process in the development of a health knowledge transfer concept for patients with heart failure.

The foundation of this work was the user-centered design process, a common method in interaction design [26]. It emphasizes the involvement of the potential end-users throughout the developmental phases to ascertain that the resulting concepts align with their needs, preferences, and experiences of the target group. This iterative approach often incorporates techniques such as user interviews, focus groups, prototyping, and usability testing to refine the design based on continuous feedback. The graphical abstract (Figure 1) provides an overview of the methodology used.

First, we used current literature and semistructured qualitative interviews to understand the context of use and derive requirements. Based on the findings, possible design solutions were drafted as a paper prototype. Thereafter, a digital high-fidelity prototype was implemented. This digital prototype was then evaluated in a study focusing on the identified requirements of the target group.

The exemplary app we used for the prototype design is the ProHerz app (ProCarement Gmbh, Germany), a medical-grade mobile app for heart failure. This app is intended to support patients in managing their health by tracking vital signs and medication intake. Additionally, the company provides a ‘CareCenter,’ where, depending on the product used, medical experts regularly monitor the patients’ health parameters and stay in personal contact via messages and phone calls.

### Stakeholder Interviews

#### Recruitment

Qualitative semistructured interviews were conducted with patients (n=9) and medical experts (n=5). This approach is a flexible data collection method that combines predefined questions with the freedom to explore emerging topics to further enhance the understanding of participants’ experiences and perspectives [27]. All patients were recruited from the ProHerz user base. Thus, all were patients with heart failure and previous experience with the ProHerz app. We chose this group because of their experience using a mobile health app for self-management. To allow for meaningful comparisons of feedback, our recruitment strategy aimed to include participants with varying levels of experience using the ProHerz app, aligning with best practices in end-user and usability testing. Medical experts were recruited through ProCarement, where they interact with the ProHerz users in their daily work. All experts were nurses with long-term experience in heart failure care. Their close exchange gives them a broad perspective on patients’ characteristics, needs, and skills.

#### Procedure

**Figure 2.**
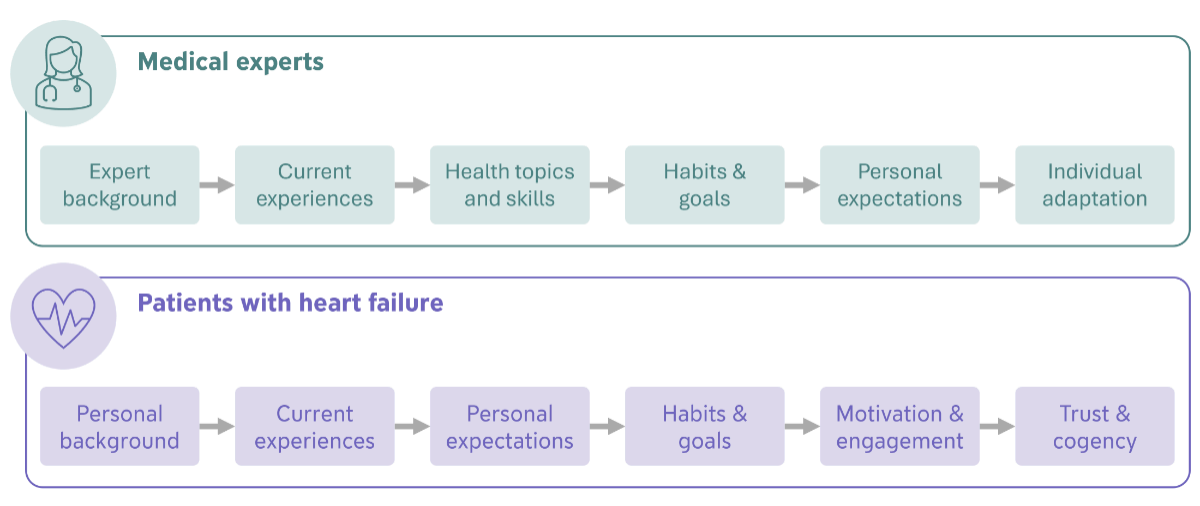
The topics included in the interview with both stakeholder groups.

The interview guidelines were based on our research aims and current literature. Both stakeholder interviews covered similar aspects (Figure 2). The guidelines aimed to identify patients’ specific needs and requirements regarding content and features within a mobile health knowledge transfer.

The interviews with the medical experts targeted explicitly at their perspective on essential knowledge and skill requirements that should be covered in a mobile health knowledge transfer concept. Besides, specific features, content presentation, and adaptation strategies for individual patient needs were discussed.

In the patient interviews, we inquired about experiences with specific features, their current interactions with the ProHerz app, and their expectations regarding knowledge transfer within a mobile health app designed for heart failure. Furthermore, the interviews included discussions on patients’ health-related routines and goals and identifying requirements for trust-building and cogency. Additionally to the interview, patients were asked to complete the European Health Literacy Survey Questionnaire (EU-HLS-Q16) to assess their health literacy level [25,26]. Through this tool, participants rate general tasks related to health literacy based on their level of difficulty.

All interviews with medical experts were conducted via Microsoft Teams. Patient interviews were conducted in person.

### Prototype Development

The development of the knowledge transfer concept was based on the findings from the interviews and related literature.

We incorporated the framework of Riegel et al to structure the topics [28]. The content categories in the prototype are oriented toward the three self-care processes for heart failure: maintenance, symptom perception, and management. Information in the prototype was structured based on the principles for effective e-learning by Clark and Mayer and the recommendations for designing health-literate mobile apps by Broderick et al [29,30]. This included, for example, dividing information into manageable units, using different types of media, and writing in a conversational style.

Additionally, we considered possible cognitive limitations of users in the context of heart failure [22,31-34]. According to the Cognitive Load Theory, a reduction of cognitive load is able to improve learning outcomes. The theory describes three types of cognitive loads in the context of learning: intrinsic, extraneous, and germane cognitive load. We aimed at reducing the extraneous cognitive load by simplifying navigation, avoiding unnecessary elements, and ensuring a clear, uncluttered interface design. The intrinsic load was managed by sequencing information into smaller units aligned with the complexity of topics. The germane load was supported by including interactive elements such as quizzes or visual aids, encouraging active engagement.

Personalizing health education to address individual needs has been shown to improve health outcomes in areas such as physical activity, nutrition, and adherence to screening recommendations [35]. In the context of health education for heart failure, users face unique challenges through varying levels of health literacy and cognitive impairments [13]. As such, a personalized approach that takes relevant individual factors into account can meet diverse needs of patients, ensuring information is both accessible and relevant [22]. For this reason, we decided to incorporate the possibility of adapting the contents based on a short survey participants answered in the beginning.

The derived themes of the interviews were brought together with findings from the health literacy assessment that additionally revealed the needs of patients. We drafted a workflow for the mobile knowledge transfer and implemented this as a low-fidelity paper prototype.

For the first usability test, we embedded the paper prototype in Useberry (Useberry User Testing Technologies IKE, Greece). Useberry is a tool to test the usability of the paper-based prototype digitally. We aimed to find usability issues as early as possible in our design. For this initial testing, we invited healthy participants through personal contact. At this stage, the priority was gathering quick insights from users who were familiar with mobile apps to identify basic usability issues before moving on to the larger-scale user testing with patients with heart failure. This reduced the burden and required time and effort of the actual patients. Test users (n=5) were asked to perform different navigation-related tasks within the prototype. Outcome measures were the task completion rate and the recorded clicks.

**Figure 3.**
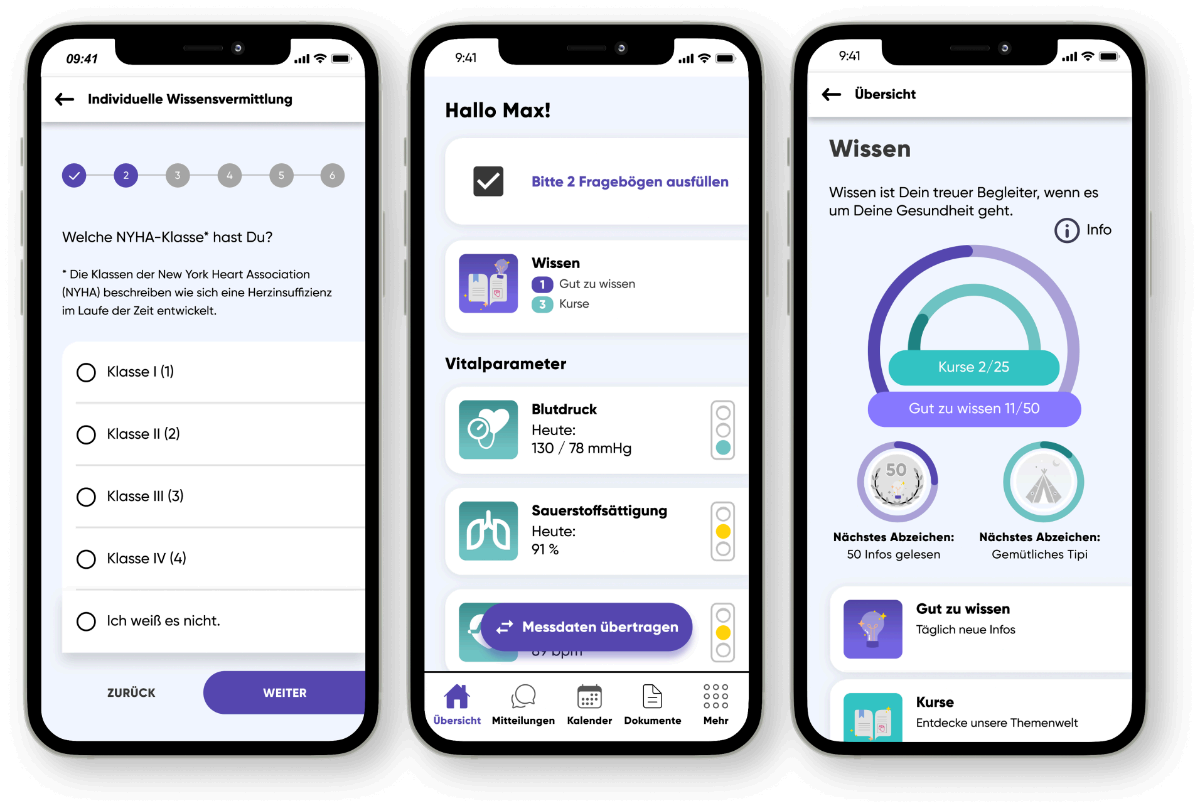
Questionnaire for individual adaptation (left), home screen of the app with the link to the knowledge section (middle), and the main page of the knowledge section (right) of the digital prototype.

**Figure 4.**
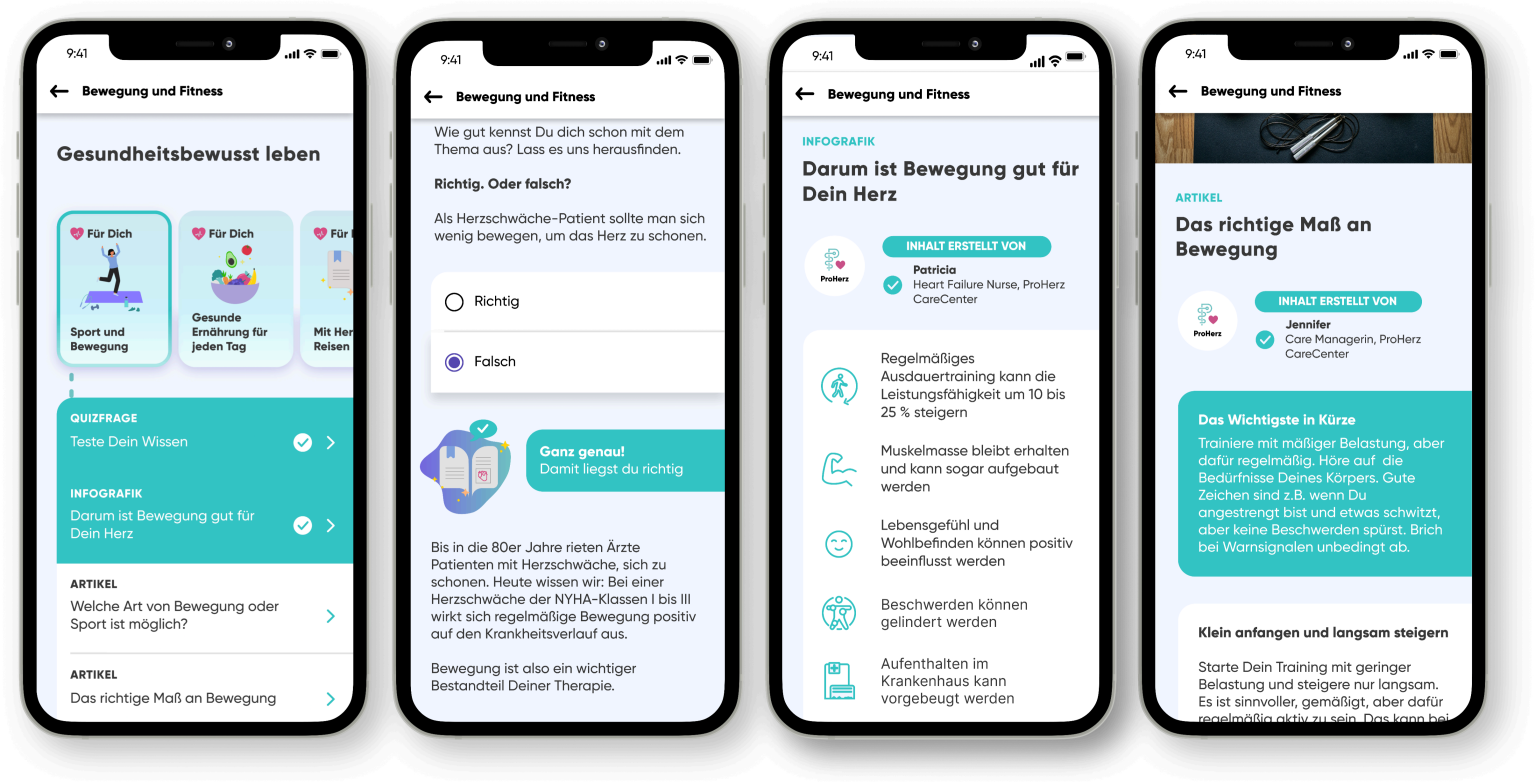
Structure of one exemplary course with (1) the overview of the content, (2) a quiz question, (3) an infographic presenting key facts, and (4) an article starting with information about the author of the text and a summary of the most important aspects of the article.

After this initial usability testing, the paper prototype was then transferred into a digital high-fidelity prototype using Figma (Figma GmbH, Germany). This prototype included an example questionnaire for individualizing the information content based on the date of diagnosis, implanted devices (pacemaker and defibrillator), smoking habits, topics of interest, the preferred media formats and the New York Heart Association (NYHA) functional class (Figure 3). The NYHA classification is a commonly used system for categorizing the severity of heart failure based on physical activity limitations caused by cardiac symptoms. It ranges from Class I (no limitations) to Class IV (highest limitations) [36]. After the questionnaire, the basic landing page of the app was presented with an additional link to the health knowledge section. The main part of our prototype was the knowledge section with an overview and link to the available courses and the short information messages. Additionally, badges for learning-related achievements can be found there. Finally, an example course was implemented on the topic of sports and exercise (Figure 4).

### Concept Evaluation

#### Recruitment

For evaluating the knowledge transfer concept, we recruited patients with heart failure (n=7), who were already experienced in using a mobile health app. Therefore, all participants were recruited from the user base of ProCarement. Four participants already took part in the previous stakeholder interviews conducted as part of this study.

#### Procedure

After being instructed about the study procedure, participants were asked to complete 11 tasks with the digital prototype. Due to feasibility reasons, patients accessed the prototype on a computer interface. This approach allowed participants to interact with the app more comfortably during the evaluation, while also enabling researchers to observe user interactions in detail and collect qualitative feedback efficiently. Tasks included small, simple tasks and more complex, time-consuming tasks. An overview of all tasks is listed in Table 1. Afterwards, semistructured interviews were conducted to obtain the participants’ overall impression and to identify features and characteristics that have the potential to improve engagement and increase health literacy.

**Table 1.** Tasks given to participants in the evaluation of the digital prototype of the health knowledge transfer concept.

No.	Description
1	Navigate to the questionnaire for individualization
2	Complete the questionnaire for individualization
3	Navigate to the knowledge section
4	Navigate to informative notifications, read the first unread notification
5	Navigate to the educational courses
6	Navigate to the course category ‘symptoms’
7	Navigate to completed courses
8	Select a course within a course category
9	Navigate to recommended courses and select a recommended course
10	Complete one educational course
11	Navigate to the overview of badges

### Analyses

All qualitative data obtained during interviews were analyzed using thematic analysis based on the approach of Braun and Clarke [37]. This method involves identifying, analyzing, and reporting patterns (themes) within the data, allowing for a rich and detailed interpretation of the participants’ experiences and perspectives. The process includes familiarizing oneself with the data, generating initial codes, searching for themes, reviewing themes, and defining and naming them to ensure a comprehensive understanding of the qualitative insights.

### Ethical Considerations

All presented substudies were approved by the institutional review board of the Friedrich-Alexander-Universität Erlangen-Nürnberg (approval no. 22‐233-S), and we obtained written informed consent from all participants. The collected data were pseudonymized after collection to ensure participant confidentiality and data protection.

## Results

### Stakeholder Interviews

#### Demographics

The stakeholder interviews with patients and medical experts were conducted in August 2022. We recruited 9 patients with a mean (SD) age of 67 (7) years. Patients received their diagnosis between 15 and 3 years prior to this study. All patients were using the ProHerz app for at least 4 months (Table 2). Seven identified as women, and 2 identified as men. Additionally, 5 medical experts were recruited (Table 3) with work experience between 6 and 11 years.

**Table 2.** Characteristics of patients with heart failure included in the stakeholder interviews.

Interviewee ID	Age (y)	Identified as	Years since heart failure diagnosis	Months of app experience with ProHerz
P1	60	female	5	9
P2	58	female	3	5
P3	75	female	3	15
P4	78	female	5	15
P5	63	female	4	16
P6	75	female	9	4
P7	69	female	15	5
P8	60	male	3	4
P9	68	male	9	6

**Table 3.** Characteristics of medical experts included in the stakeholder interviews.

Interviewee ID	Identified as	Expert experience	Years of work experience with patients with heart failure
Expert 1	female	Heart failure nurse, licensed practical nurse	11
Expert 2	male	Licensed practical nurse	6
Expert 3	female	Licensed practical nurse	6
Expert 4	female	Bachelor of Science in Nursing, specialist in anesthesia and intensive care	9
Expert 5	male	State-qualified nurse	8

#### Thematic Analysis

We derived five themes relevant to a knowledge transfer concept from the stakeholder interviews with patients with heart failure and medical experts.

##### Individual Adaptation of Health Information

The interviews revealed a positive perspective towards the adaptation of content and push notifications to the different levels of knowledge of the users (4/5 experts, 2/9 patients). Our results suggest individualization based on different information such as the NYHA class, walking distance, app usage duration, physical condition age, prior knowledge, or personal preferences that could be determined for example through a quiz within the app.

##### Relevant Health Topics for Patients With Heart Failure

**Figure 5.**
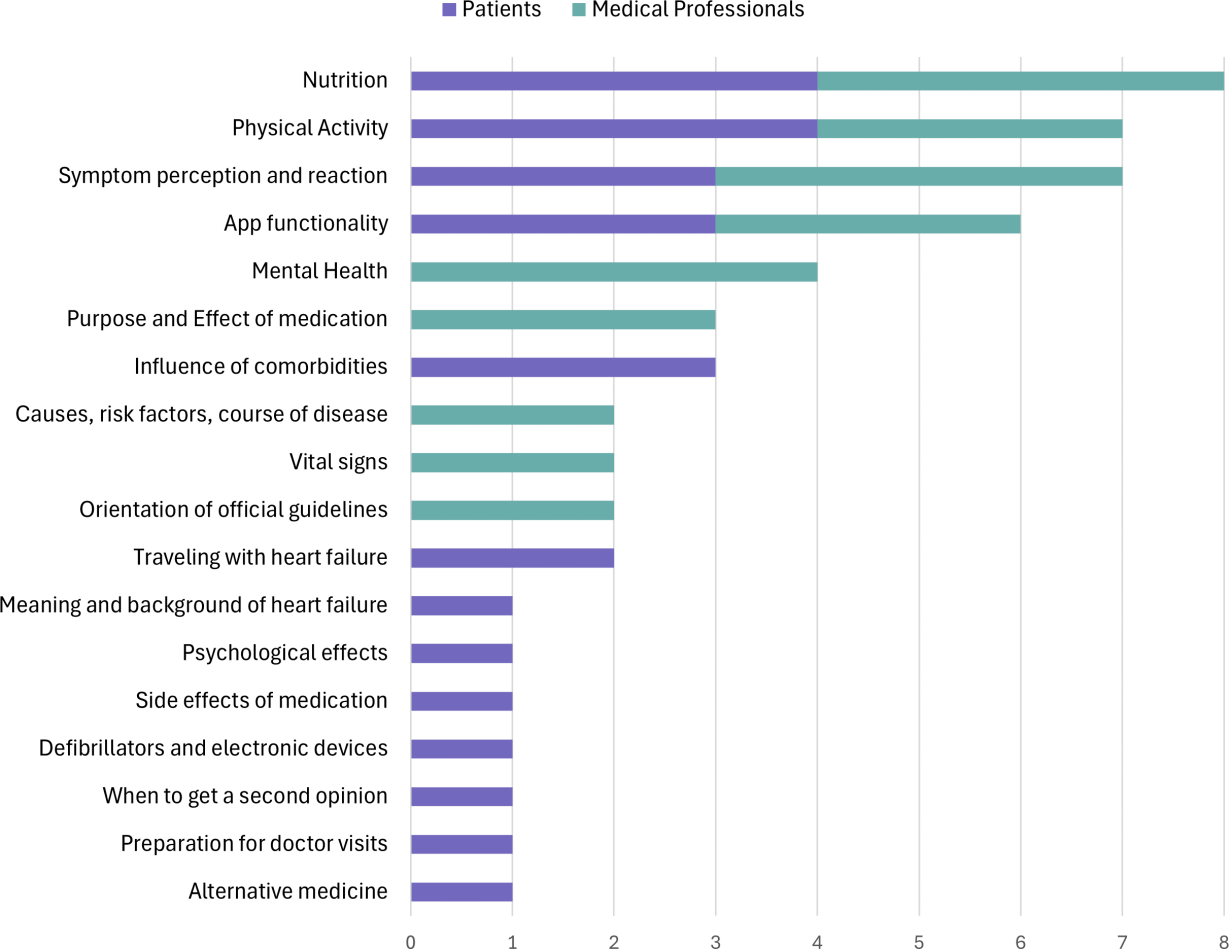
Health topics mentioned in the stakeholder interviews with patients and medical experts and that were considered relevant to be included in a digital knowledge transfer concept.

Several relevant health topics were identified within the stakeholder interviews (Figure 5). Health topics most requested by both patients and medical experts included nutrition (4/5 experts, 4/9 patients), physical activity (3/5 medical experts, 4/9 patients), symptom perception and reaction (4/5 medical experts, 3/9 patients), and app functionality (3/5 medical experts, 3/9 patients).

##### Diversity of Media Formats

Most of the interviewed patients (7/9 patients) preferred information in text format. For example, 1 participant stated, “you can read it two or three times if you say it’s something that’s difficult to absorb, maybe a text would be better” (P5, translated). Additionally, videos were perceived as useful by all medical experts but only desired by 2 patients. Related to the presentation of the information, patients mentioned using fewer technical terms and more simple language (2/9 patients) and shortly highlighting the most important aspects (2/9 patients). This can support easy comprehension of contents, which was also mentioned as a factor for the trustworthiness of health information (3/9 patients).

##### Promotion of Motivation, Engagement, and Joy

Medical experts (4/5 experts) suggested a reward system to increase engagement with the information contents. However, it should be ensured that this reward system is suitable for the specific target group of users (2/5). Three patients also mentioned the wish to receive positive feedback for progress within the app. Personal interest in the content was also mentioned (3/9) to have a positive influence on engagement.

##### Credibility and Trust

From the interviews with patients, we identified several factors related to the credibility of information. Positively perceived was the specification of authors (3/9 patients), especially if the authors are physicians (2/9 patients) or authors with an academic background (2/9 patients). In addition, the provider of the mHealth app should have a good reputation (3/9 patients). Factors restraining trust and credibility could be advertising (1/9 patients), authors without medical background (1/9 patients), or irrelevant information (1/9 patients).

### Health Literacy

Health literacy levels based on the EU-HLS-Q16 were between 10 and 16 with an average (SD) score of 13.6 (2.1). Participants mostly had difficulties deciding on preventive actions based on media information (5/9 patients) and determining whether information about health risks in media is trustworthy (4/9 patients).

### Evaluation of the Digital Prototype

#### Demographics

We evaluated the proposed digital prototype of the knowledge transfer concept with 7 patients with heart failure (Table 4). The mean (SD) age was 65 (6) years. Patients received their diagnosis between 2 and 24 years prior to this study. All patients were using the ProHerz app for at least 5 months. Four participants identified as women, and 3 as men. This group was less diverse in terms of ProHerz experience, which was a result of recruiting participants based on availability and willingness to participate.

**Table 4.** Characteristics of patients included in the evaluation study of the knowledge transfer concept.

Interviewee ID	Age (y)	Gender	Years since heart failure diagnosis	Months of app experience
P1	60	female	5	9
P2	58	female	3	5
P3	75	female	3	15
P5	63	female	4	16
P10	59	male	9	15
P11	71	male	2	15
P12	69	male	24	10

#### Usability

The mean task completion rate was 84%; 5 of the 11 tasks given during the study were completed by all participants. The lowest completion rate occurred with task 4, which was completed by 4 of 7 participants (Table 5).

**Table 5.** Task completion rate for all tasks performed by the participants within the usability testing.

No.	Description	Completion rate (%)
1	Navigate to the questionnaire for individualization	100
2	Complete the questionnaire for individualization	100
3	Navigate to the knowledge section	100
4	Navigate to informative notifications, read the first unread notification	57
5	Navigate to the educational courses	71
6	Navigate to the course category ‘symptoms’	71
7	Navigate to completed courses	71
8	Select a course within a course category	71
9	Navigate to recommended courses and select a recommended course	86
10	Complete one educational course	100
11	Navigate to the overview of badges	100

#### Interview Results

The thematic analysis of the semistructured interviews with patients within the evaluation of our proposed concept resulted in 3 main themes.

##### Knowledge Transfer

In the evaluation of the digital prototype, the majority of participants (4/7 patients) classified the prospective range of information as valuable. The organization and structure of contents received positive feedback from most participants (5/7 patients). Specifically, the clear arrangement of information (3/7 patients) and the definition of specific categories (2/7 patients) were highlighted. Patients also perceived the individual adaptation as positive, because personally irrelevant content would be hidden (4/7 patients).

To further improve the concept, participants indicated the necessity to update contents in response to potentially evolving needs over time due to lifestyle or health condition changes (2/7 patients).

##### Motivation and Engagement

A majority (5/7 patients) expressed an intention to utilize the course content in the future. Notably, reminders about feasible health-promoting measures were identified as a motivational factor by 3 out of 7 patients, with a specific emphasis from 2 participants on messages related to physical activity.

Concerns were raised about the potential negative impact of irrelevant, repetitive, or overly frequent notifications (4/7 patients). Conversely, aligning information with individual interests was identified as a key factor in promoting joy and sustaining engagement among users.

Patients expressed joy while answering the quiz questions, which may have contributed to a more engaging experience (2/7 patients). There was a suggestion to place quiz questions at the end of a course to assess the comprehension of the content effectively.

Participants generally responded positively to medical staff monitoring of progress, considering it a supportive element in their engagement with the course. However, 1 participant viewed social comparison negatively. Furthermore, intangible rewards, such as badges, were not universally well-received, with 1 out of 7 participants expressing a negative opinion, stating that it is solely virtual and not a real reward (P11).

##### Ease of Use

Out of 7 patients, 3 patients explicitly mentioned the app as easy to use. Other participants did not comment on this aspect, neither positively nor negatively. Concerns regarding the ease of use included potential initial overload when first using the app and potential individual difficulties with the technology and navigation.

## Discussion

### Principal Findings and Implications

In this study, we developed a concept for mobile health knowledge transfer for patients with heart failure. We grounded this concept on the requirements of patients with heart failure and current theoretical frameworks. From the patient and caregiver perspectives, we derived five central themes for a health knowledge transfer concept.

Our findings contribute to the understanding of user preferences and content organization in digital health interventions targeted at users with heart failure. The integration of diverse methodologies, including qualitative interviews, usability testing, and participant feedback, fortifies the study’s comprehensiveness, providing insights into user engagement, health literacy promotion, and the practical implications for digital health interventions. The results of this study represent an important step toward the development of a mobile health knowledge transfer tool for heart failure patients. By identifying relevant features and characteristics, this work provides a foundation for further refinement and testing, ultimately aiming to support health literacy and improve patient outcomes. It becomes evident that a concept should focus on individualization, topic relevance, media diversity, motivation strategies, and trust-building mechanisms to optimize its impact on health literacy and engagement of patients with heart failure. Based on the findings, identified in the stakeholder interviews, we developed a concept for knowledge transfer in a digital health app for patients with heart failure.

We evaluated our concept with a digital prototype and identified features and characteristics that are perceived as supportive in increasing health literacy, that can promote motivation, engagement, and joy, and that are able to convince patients of a mobile health knowledge transfer. Most prominent was the wish for content that is individually adapted and balanced regarding the topics covered as well as media formats used to present those topics.

The results of the prototype evaluation presented both encouraging feedback and areas for refinement. Participants highlighted the app’s structured design and ease of navigation, emphasizing that these features facilitated a straightforward user experience and enhanced the clarity of the presented information. The quiz elements, in particular, were frequently mentioned as engaging and motivating, showcasing the potential of interactive components to sustain user interest and reinforce learning. These positive responses suggest that the app effectively addressed some of its primary goals, including improving knowledge transfer and supporting patient engagement.

Conversely, the feedback also identified challenges that should be addressed in future iterations. Participants expressed concerns of getting overwhelmed, potentially hindering comprehension. This may reflect concerns about managing excessive information or complex interactions within the app, particularly in the context of their health condition. For users with chronic conditions like heart failure, the introduction of too much content or functionality in a single interface could exacerbate feelings of being overwhelmed and lead to disengagement or frustration [38]. Furthermore, badges and achievements were mentioned to be not motivating despite research showing the positive effects of such gamification elements [39,40]. Older users or those less familiar with gamification might find such features irrelevant or even patronizing if they do not see clear value in them. Other gamification elements such as progress bars, interactive storylines, or more useful rewards may be more effective in motivating users. Therefore, future iterations could explore the inclusion of a simple setup and customization menu to enable or disable features such as gamification and other user-preferred functionalities.

Accessibility is a critical consideration in designing digital health systems to ensure they meet the needs of diverse users. While none of the participants in our study raised concerns or reported difficulties with accessibility, indicating that the system was well-received by the target population, it is important to account for potential challenges in broader user groups. Features such as larger fonts, high-contrast visuals, simplified navigation, and optional audio support can further enhance usability, particularly for individuals with age-related changes in vision, hearing, or varying levels of digital literacy.

The high rates of completion for most tasks suggest that the interface and navigation design are intuitive, enabling users to locate and interact with the desired functions efficiently. This aligns with the positive feedback received during the interviews conducted within the evaluation. However, with a few tasks having a lower completion rate, some aspects of the design need further refinement in future iterations. Factors such as differences in technology familiarity, cognitive load, or previous experience with the ProHerz app could have contributed to these discrepancies. Interactive tutorials to onboard users and a further adaptation of content may be able to better account for different requirements, levels of experience, or cognitive abilities. In particular, task 4 with a completion rate of only 57% was the most problematic task for participants. The participants were not able to locate the button to navigate to the informative notifications. The reason for this is most likely the design of the progress bar on the same page (Figure 3, right) that users expected to be the button to the next page. Ensuring more consistent visual cues for interactive elements could further reduce the potential for misinterpretation and enhance the overall user experience.

### Comparison With Prior Work

The need for individualization is in line with the findings of Giordan et al [41], calling for further customization especially related to the educational level and digital literacy of users. Our results additionally highlight the need for customization regarding personal interests and lifestyle and the importance of regular adaptation to changing circumstances in the life and knowledge of users. Future applications require new strategies to individually adapt educational content that can change over time. This can include regularly soliciting user feedback through surveys, using quizzes to assess understanding, or adaptive learning algorithms based on user behavior within the mobile app [42].

The topics covered in our knowledge transfer concept are in line with the recommendations of the European Society of Cardiology. They included information about heart failure, symptom monitoring, self-management, medication, fluid intake, implantable devices, physical activity, nutrition, smoking, sleep mental health, and traveling [43]. The information was presented using different media formats, which relates to current literature emphasizing the benefits of this approach. A variety of formats can support users with different health literacy levels and can deepen the learning effects [29,43,44].

### Strengths and Weaknesses

Several limitations apply to this study. Firstly, the participant pool featured a restricted number of individuals, all German-speaking and residents in Germany. Moreover, participants were well-acquainted with the ProHerz app, potentially influencing their perspectives and feedback, especially regarding the ease of use. Given the advanced health literacy levels of the participants, our findings may not comprehensively represent the needs of individuals with lower health literacy. The initial usability testing was conducted with a convenience sample due to limited patient availability; therefore, results from this group need to be interpreted with caution. During the evaluation phase, 4 participants already were part of the stakeholder interviews in the first phase of this study due to limited participant availability. Lastly, all participants accessed the app on a laptop rather than a mobile phone, introducing a potential discrepancy, as some users reported difficulties with computer usage and the overall interaction experience may be different. These limitations underscore the necessity for cautious interpretation and highlight avenues for future research refinement.

Our study combines several strengths: we were the first to conceptualize a mobile health knowledge transfer within a user-centered design approach. By prioritizing patient needs and requirements, and involving healthcare professionals to ensure relevance and meaningfulness, the study sets a precedent in fostering impactful and patient-centric digital health interventions. The incorporation of theoretical frameworks for mobile health education enriches the conceptual foundation, contributing to the overall robustness of the study and its potential impact on the enhancement of patient-centered care.

### Conclusions

We were the first to develop a mobile health knowledge transfer for heart failure grounded on theoretical frameworks within a user-centered design process.

By addressing individual health literacy levels, cognitive challenges, and personal preferences, this knowledge transfer concept can help patients better understand and manage their condition. The active involvement of healthcare professionals ensures that the resulting intervention is meaningful and aligned with relevant healthcare aspects. The findings also emphasize a discrepancy between what healthcare professionals perceive as important for patients and what patients themselves prioritize, particularly when it comes to identifying relevant healthcare topics. Our results are paving the way to more personalized and effective mobile health education for individuals managing heart failure. The concept can be used as a foundation for other digital platforms and mobile health apps to provide tailored educational content and supporting clinicians in delivering consistent, patient-specific information in home settings.

Future research opportunities should focus on longitudinal studies to assess the sustained impact of this knowledge transfer concept on health literacy, self-care behavior, and patient outcomes. In this context, validated usability scales alongside user interviews should be incorporated during iterative development processes, while also addressing potential biases in design and testing to ensure more robust and generalizable findings. Additionally, possible strategies for better individualization of health information should be further explored. To better understand the impact of personalization, future studies could incorporate a comparison of user experiences before and after customizing the app, providing valuable insights into how individualization influences usability and user satisfaction. This presents an opportunity to investigate adaptive learning algorithms that dynamically tailor content based on user progress and preferences.

## Abbreviations

NYHA: New York Heart Association
